# Dendritic Cell-Targeted pH-Responsive Extracellular Vesicles for Anticancer Vaccination

**DOI:** 10.3390/pharmaceutics11020054

**Published:** 2019-01-27

**Authors:** Hyuk Lee, Hongsuk Park, Hyeong Sup Yu, Kun Na, Kyung Taek Oh, Eun Seong Lee

**Affiliations:** 1 Department of Biotechnology, The Catholic University of Korea, 43 Jibong-ro, Bucheon-si, Gyeonggi-do 14662, Korea; ahld1421@naver.com (H.L.); skleelsk@naver.com (H.S.Y.); kna6997@catholic.ac.kr (K.N.); 2 Division of Endocrinology, Metabolism & Lipid Research, Washington University School of Medicine, Saint Louis, MO 63110, USA; hongsuk.park@wustl.edu; 3 College of Pharmacy, Chung-Ang University, 84 Heukseok-ro, Dongjak-gu, Seoul 06974, Korea; kyungoh@cau.ac.kr

**Keywords:** extracellular vesicles, pH-responsive, dendritic cells, toll-like receptor 4 signaling, anticancer vaccine

## Abstract

Immunotherapy can potentially treat cancers on a patient-dependent manner. Most of the efforts expended on anticancer vaccination parallel the efforts expended on prototypical immunization in infectious diseases. In this study, we designed and synthesized pH-responsive extracellular vesicles (EVs) coupled with hyaluronic acid (HA), 3-(diethylamino)propylamine (DEAP), monophosphoryl lipid A (MPLA), and mucin 1 peptide (MUC1), referred to as HDEA@EVAT. HDEA@EVAT potentiated the differentiation and maturation of monocytes into dendritic cells (DCs) and the priming of CD8^+^ T-cells for cancer therapy. MPLA and HA enabled HDEA@EVAT to interact with the toll-like receptor 4 and the CD44 receptor on DCs, followed by endosomal escape, owing to the protonation of pH-sensitive DEAP on the EV in conjunction with MUC1 release. The MUC1 was then processed and presented to DCs to activate CD8^+^ T-cells for additional anticancer-related immune reactions. Our findings support the anticancer vaccine activity by which HDEA@EVAT expedites the interaction between DCs and CD8^+^ T-cells by inducing DC-targeted maturation and by presenting the cancer-associated peptide MUC1.

## 1. Introduction

Human health is predominantly regulated by the immune system that distinguishes the non-self from the self. Given that cancer cells originate from our own cells, it is difficult to manage uncontrollable cell growth based on immune reactions. Recently, cancer immunotherapy has sparked considerable interest in cancer treatment by enhancing the anticancer immune system in the body using engineered T-cells and chimeric antigen receptors (CARs) [1,2,3]. The CAR T-cell technology has shown unprecedented success in the long-term efficacy and the therapeutic response rates of cancer therapies [4,5]. In addition, introducing immune checkpoint inhibitors, such as anti-cytotoxic T-lymphocyte-associated antigen 4 (anti-CTLA4), and cancer-targeted vaccines have expedited the field of therapeutic anticancer approach by boosting anticancer immune functions. In particular, melanoma-aimed vaccines containing adenovirus-transduced dendritic cells (DCs) facilitated T-cell-involved immunity [6,7,8,9]. These types of therapeutic approaches are definitely attractive and smart because they stimulate and use the body’s natural defenses to effectively attack cancer cells, unlike conventional surgery, radiotherapy, or chemotherapy.

Toll-like receptor (TLR) agonists, such as monophosphoryl lipid A (MPLA), have been introduced into vaccine constructs to expedite immune responses against cancer cells. The immunomodulator, MPLA, is a non-toxic lipopolysaccharide (LPS) derivative, and favorably recognizes and binds to TLR4 receptors on the cell surface, followed by the transduction of TLR4 signaling [10,11]. Beyond the provision of the essential signal for naive T-cell activation, the CD86 overexpression indicates the maturation of DCs. In other words, the MPLA/TLR4 signaling induces the maturation along with CD86 upregulation [12,13]. The mature DCs secrete critical inflammatory cytokines, including tumor necrosis factor-α (TNF-α) [14,15]. While immature DCs reside in non-lymphoid tissues, mature DCs exhibit upregulated CD86 and major histocompatibility complex (MHC) expressions, and they move to secondary lymphoid organs as bona fide antigen-presenting cells to interact with T-cells to mediate adaptive immune responses [16]. 

Extracellular vesicles (EVs), including exosomes, microvesicles, and apoptotic bodies, are lipid bilayer constructs that originate from the cell membrane, and act as bioactive cargos for cell-to-cell interplay and biological delivery. In recognition of the growth of their versatile characteristics, significant interest has focused on the encoding of biological payloads in EVs. More specifically, EVs can effectively embed and carry endogenous components, such as RNAs, peptides, and lipids, as well as exogenous drugs and viruses [17,18,19]. In response to the growing attention to exploring cancer vaccine, Dexo (EVs from DCs) was identified and its application established the foundation for EV-based cancer immunotherapy [20,21]. In addition, even though the typical non-viral delivery vehicle, liposome, has intrinsic problems involving toxicity and immune activation by virtue of their foreign nature, patient-derived EVs are immunologically inert. Most of the efforts attributed to the EV characteristics in drug delivery systems parallel the efforts expended on liposomal features in nanomedicine approaches. However, EVs can more favorably deliver the biomolecules that are difficult to load to recipient cells for modification unlike their synthetically synthesized liposomal counterparts [17,22]. 

In the preparation of drug carriers, the conjugations of pH-responsive moieties with backbone molecules have stimulated various research studies on therapeutic applications [23,24,25,26,27,28]. In particular, protonation of amines in pH-sensitive molecules results in buffering inside endosomes after they are endocytosed followed by osmotic swelling and endosomolysis. The “proton sponge” effect leads to the endosomal escape of drug delivery agents from lysosomal degradation together with the release of therapeutic payloads from the delivery devices [29,30,31,32]. Moreover, to promote the selective interaction between a targeted cell and a delivery tool, the ligand–receptor system can be exploited further. Because CD44 receptors are overexpressed on DCs [33,34], hyaluronic acid (HA) is a potential candidate as a DC ligand conjugate for receptor-mediated endocytosis [24,25,35,36]. 

In a previous study, we developed the EV-entrapped construct with the pH-responsive 3-(diethylamino)propylamine (DEAP) and the CD44 ligand HA, referred to as HDEA@EV, to deliver the anticancer drug doxorubicin [25]. In this study, the MPLA was used as a TLR4 ligand, and mucin 1 (MUC1) was used as a cancer-involved antigen. MPLA and TLR4 were then attached to prepare the HDEA@EV-based anticancer vaccine (HDEA@EVAT). The HDEA@EVAT has two HA and MPLA ligands for the CD44 receptor and TLR4, respectively, enabling it to bind to DC efficiently and tightly. After the cellular uptake, HDEA@EVAT ruptured the endosomes owing to the “proton sponge” effect by DEAP, discharging the MUC1 antigen. MUC1 is involved in the activation of unprimed T cells that turn into interferon gamma (IFN–γ)-producing activated T-cells [37,38]. Overall, this EV-based reductionist approach to activate immune cells could be used as a treatment for various cancer diseases.

## 2. Materials and Methods

### 2.1. Materials

Hyaluronic acid (HA, MW = 4.8 kDa), 3-(diethylamino)propylamine (DEAP), *N*-hydroxysuccinimide (NHS), *N*,*N*′-dicyclohexylcarbodiimide (DCC), triethylamine (TEA), deoxycholic acid (DOCA), 4-dimethylaminopyridine (DMAP), pyridine, dimethyl sulfoxide (DMSO), sodium tetraborate (Na^2^B^3^O^7^), adipic acid dihydrazide (ADH), monophosphoryl lipid A (MPLA), red blood cell lysis buffer, acridine orange hemi (zinc chloride) salt, dihydrochloride (DAPI), and paraformaldehyde, were obtained from Sigma–Aldrich (St. Louis, MO, USA). Chlorin e6 (Ce6) was purchased from Frontier Scientific Inc. (Logan, UT, USA). Human MUC1 BP 16 (GVTSAPDTRPAPGSTA > 95% purity) (MUC1) was purchased from Peptron Inc. (Daejeon, Korea). The lipid quantification kit was purchased from Cell Biolabs Inc. (San Diego, CA, USA). A micro BCATM protein assay kit was purchased from Thermo Fisher Scientific Inc. (Walthan, MA, USA). RPMI 1640 medium, Dulbecco’s modified Eagle medium (DMEM) medium, fetal bovine serum (FBS), ethylene diamine tetra-acetic acid (EDTA), penicillin, trypsin, and streptomycin were purchased from Welgene Inc. (Seoul, Korea). Exo-depleted FBS was obtained from System Biosciences Inc. (Johnstown, PA, USA). Moreover, recombinant murine GM–CSF (GM–CSF) and recombinant murine IL–4 (IL–4) were purchased from Pepro Tech Inc. (Seoul, Korea). Cell Counting kit-8 (CCK–8) was obtained from Dojindo Molecular Technologies Inc. (Santa Clara, CA, USA). FITC rat anti-mouse CD86 clone GL1 (FITC–CD86 antibody) was purchased from BD Biosciences (San Jose, CA, USA). Wheat germ agglutinin Alexa Fluor^®^ 488 conjugate (WGA–Alexa Fluor^®^ 488) was bought from Thermo Fisher Scientific Inc. (Walthan, MA, USA). Mouse TNF alpha ELISA kit and mouse interferon gamma ELISA Kit (IFNG) were purchased from Abcam Plc (London, UK). The CD8a_+_ T-cell isolation kit was bought from Miltenyi Biotec (San Diego, CA, USA).

### 2.2. Synthesis and Characterization of HDEA@EVAT

HDEA@EV (HA and DEAP-anchored EV) was prepared as previously reported [25]. Briefly, EVs were harvested from the culture medium of mouse macrophage RAW 264.7 cells. After consecutive centrifugation steps, purified EVs were collected. Meanwhile, HA was conjugated with DEAP to synthesize HA–*g*–DEAP (HDEA). To synthesize HDEA@EVAT, HDEA (100 μg), MPLA (10 μg) and MUC1 (10 μg) were separately dissolved in dimethyl sulfoxide (DMSO, 200 μL), and were both transferred to phosphate buffered saline (PBS) (10 mL) which contained EVs (100 μg). Subsequently, the mixture was sonicated using the tip sonicator vcx–130 with cv–18 (Newtown, CT, USA) with 20% amplitude and six cycles with 30 s on/150 s off periods, followed by incubation at 37 °C for 60 min. The solution was filtered with a 0.22 μm membrane and was then ultracentrifuged at 100,000× *g* at 4 °C for 70 min to separate untrapped HDEA, MPLA, and MUC1 moieties. The pellet was resuspended in PBS (1 mL) followed by lyophilization to obtain HDEA@EVAT. To make pH-irresponsive HDOC@EVAT, HA was grafted with DOCA for HDOC preparation, as previously described [25], and HDOC (50 μg), MPLA (10 μg), and MUC1 (10 μg) were encoded in EV. HDEA@EVA was produced using HDEA (100 μg) and MUC1 (10 μg), HDOC@EVA was produced using HDOC (50 μg) and MUC1 (10 μg), EVAT was prepared using MPLA (10 μg) and MUC1 (10 μg), and EVA was produced using MUC1 (10 μg).

To identify the content of encoded HDEA or HDOC in EVs, fluorescent Ce6 was incorporated in EVs and the fluorescence intensity was measured at λ_ex_ 450 nm and λ_em_ 670 nm using a fluorescence spectrofluorometer (RF–5301PC, Shimadzu, Japan). In addition, to calculate unloaded MPLA or MUC1, EVs were ultracentrifuged (100,000× *g*, 4 °C), and the supernatant was then collected followed by the measurement of lipids or proteins using the lipid quantification kit or the Micro BCA^TM^ protein assay kit, respectively. The encoding amounts (%) of HDEA, HDOC, MPLA, or MUC1, were determined as the weight percentages of HDEA, HDOC, MPLA, or MUC1 in EVs.

Hydrodynamic sizes and the zeta potentials of EVs (equivalent to 30 μg/mL EV) were analyzed using a Zetasizer 3000 instrument (Malvern Instruments, Westborough, PA, USA).

### 2.3. MUC-1 Release Profile of HDEA@EVAT

EVs (equivalent to 50 μg/mL MUC1) dispersed in PBS (3 mL) were transferred to a dialysis membrane (Spectra/Por^®^ MWCO 50 K). The dialysis membrane bag was sealed and was subsequently immersed in a vial that contained fresh 15 mL PBS. The discharge of MUC1 was performed with mechanical shaking (100 rpm) at 37 °C for 48 h. A small volume of the release medium was extracted at each time point and was analyzed by monitoring the MUC1 using the Micro BCA^TM^ protein assay kit.

### 2.4. Animal Care

Female BALB/c mice (total 20 mice, 6–8 weeks-old, Orient Bio Inc., Seoul, Korea) were used to isolate and harvest DCs. All animals and biological samples used in this work were implemented in accordance with the procedures approved by the Institutional Animal Care and Use Committee (IACUC) at the Catholic University of South Korea. 

### 2.5. Dendritic Cell (DC) and Monocyte Cultures

First, a total of 20 mice were euthanized through carbon dioxide treatment. Then, monocytes were isolated from the bone marrow tissue of the mouse femur. To purify the monocytes, they were filtered using a cell strainer (40 μm, SPL Life Sciences Inc., Pocheon-si, Korea) and centrifuged at 1000 rpm for 5 min. The pellet was incubated with red blood cell lysis buffer (1 mL) at 4 °C for 1 min followed by centrifugation at 1000 rpm for 5 min with the use of additional PBS. The pellet was harvested and cultured in RPMI–1640 medium supplemented with GM–CSF (20 ng/mL), IL–4 (10 ng/mL), 10% FBS, and 1% P/S (collectively referred to as conditioned RPMI–1640 medium). After one day, the media were changed with fresh conditioned RPMI–1640 media. Cells were maintained incubated for an additional six-day period. At that time, cells differentiated into DCs. For monocyte cultures, cells were maintained in an RPMI–1640 medium which was supplemented with 10% FBS and 1% P/S. 

### 2.6. In Vitro Endocytosis Study

EVs were labeled with Ce6 to monitor the cellular entry. EVs (equivalent to 30 μg/mL EV) were dispersed in the RPMI–1640 media, and they were treated with DCs at 37 °C for 4 h. After washing the DCs three times using PBS, fluorescence intensity and mean fluorescence intensity (MFI) were analyzed using FACSCalibur^TM^ (10^4^ repeats per experiment, Becton Dickinson, Franklin Lakes, NJ, USA). For qualitative imaging, DAPI (5 μg/mL, 1 mL, 5-min treatment) and WGA-Alexa Fluor^®^ 488 (5 μg/mL, 1 mL, 5 min treatment) were used to stain cell nuclei and cell membranes, respectively. The cells were fixed with 3.7% formaldehyde for confocal laser scanning microscopy (CarlZeiss LSM710, Oberkochen, Germany).

### 2.7. In Vitro DC Maturation Analysis

To determine whether EVs induced the maturation of DCs, FITC–CD86 antibody immunostaining was used [12,13]. DCs were incubated with EVs (equivalent to 30 μg/mL EV) for 24 h, and they were then washed three times using PBS. The FITC–CD86 antibody (10 μg/mL, 1 mL) was treated for 30 min, and PBS (4 mL) was added to dilute and disperse the samples. Fluorescence intensity and MFI were measured using FACSCalibur^TM^. 

To determine the DC maturation, the secreted TNF–α was measured [14,15]. To this end, DCs were treated with EVs (equivalent to 30 μg/mL EV) for 24 h. This was followed by the harvesting of the cells and medium. The solution was centrifuged at 1000 rpm for 5 min and was filtered using a 0.22 μm membrane to remove the cells and EVs. The TNF–α level was measured using the mouse TNF alpha enzyme-linked immunosorbent assay (ELISA) kit. 

### 2.8. CD8^+^ T-Cell Sorting

CD8^+^ T-cells were prepared from BALB/c mouse spleens to evaluate the ability of EV-stimulated DCs as antigen-presenting cells. The spleens were collected, homogenized, and transferred to RPMI–1640 media. After filtering the solutions with a cell strainer, they were centrifuged at 1000 rpm for 5 min. The pellet was treated with red blood cell lysis buffer (1 mL) at 4 °C for 1 min, and PBS (9 mL) was then added to dilute them. Subsequently, they were centrifuged at 1000 rpm for 5 min. The pellet was collected and suspended in PBS (10^8^/mL). The CD8a^+^ T-cell isolation kit (200 μL/10^8^ cell) was applied for 10 min, followed by CD8^+^ T-cell sorting in the presence of a magnetic field (VarioMACS separator, Miltenyi Biotec, San Diego, CA, USA). 

### 2.9. In Vitro Analysis of DC Antigen Presentation

After interaction between mature DCs and CD8^+^ T-cells, IFN–γ level was measured to assess the antigen-presenting capacity of DCs [37,38]. DCs were incubated with EVs (equivalent to 30 μg/mL EV) for 24 h for maturation. Subsequently, the medium was changed, and fresh RPMI–1640 media and CD8^+^ T-cells were added to interact with the mature DCs for 24 h. The cell number ratio of mature DCs to CD8^+^ T-cells was 1:3. The cells were harvested and cells and media were centrifuged at 1000 rpm for 5 min. This was followed by filtration (0.22 μm membrane) to remove cells and EVs. IFN–γ obtained from the filtrate was measured using the mouse interferon gamma ELISA kit. 

### 2.10. Statistics

All the data were analyzed using Student’s *t*-test or ANOVA at a significance level of *p* < 0.01 (**).

## 3. Results and Discussion

### 3.1. Preparation and Characterization of HDEA@EVAT

**Figure 1:**
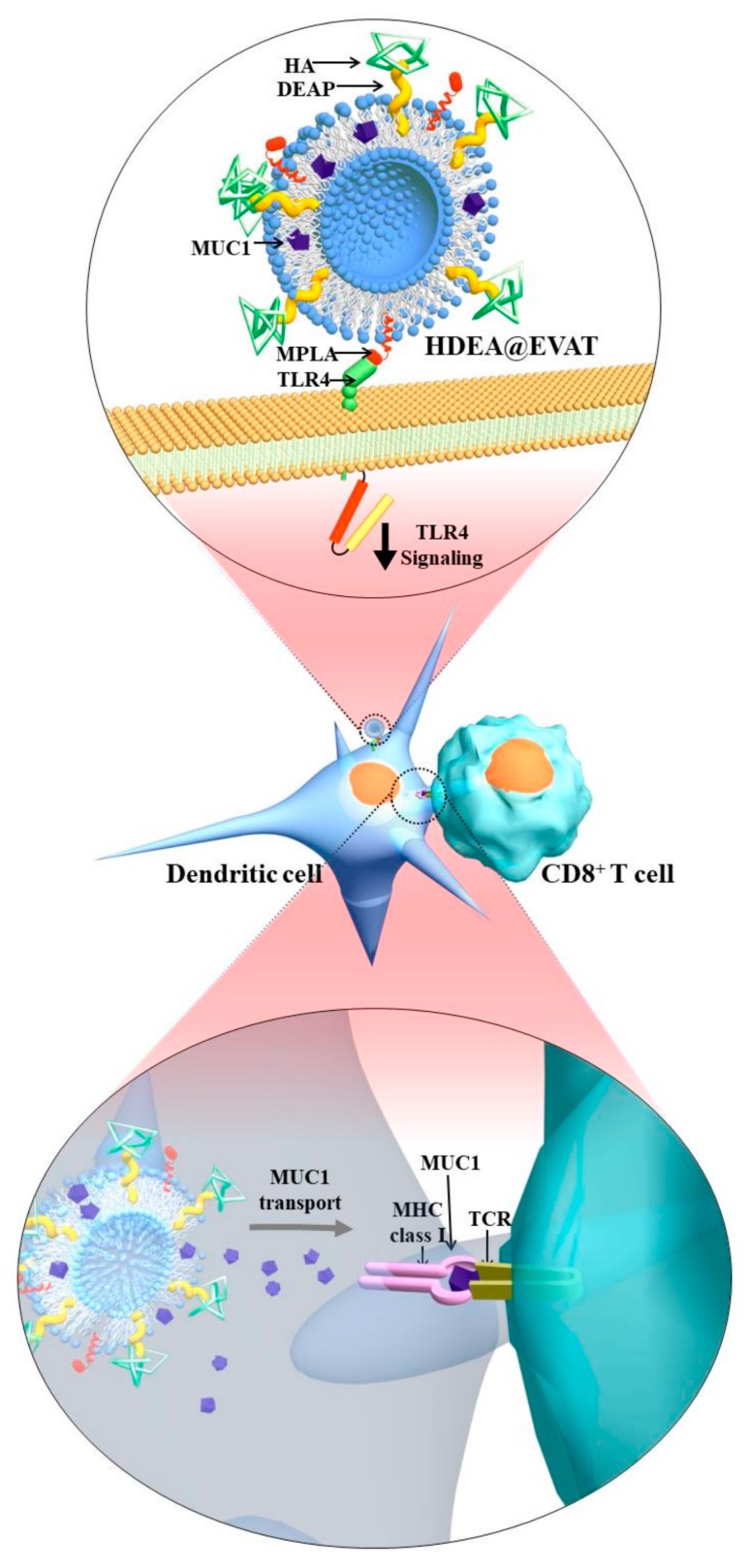
Schematic illustration of dendritic cell (DC)-targeted pH-responsive HDEA@EVAT and the intracellular process for CD8^+^ T-cell activation.

DC-based vaccination has become the principal approach to anticancer therapy because it yields strong T-cell responses. To capitalize on this technique’s advantage, we synthesized DC-targeted therapeutic nanoparticles. The HDEA@EVAT nanoparticle contains MPLA, a TLR4 ligand expressed on DCs. Correspondingly, the ligand–receptor interaction results in the maturation of DCs. In the meantime, the HA in HDEA@EVAT leads to CD44 receptor-mediated endocytosis of HDEA@EVAT into DCs, followed by the destabilization due to the pH-responsive HDEA moiety inside the endosomes. Based on the endosomal pH conditions, HDEA@EVAT elicits endosomal rupture and disintegrates. As a result, cancer-involved antigen MUC1 is released into the cytosol of DCs. DCs then processed the MUC1 using the proteasome and present it on class I MHC. The MUC1 with class I MHC is recognized by the T-cell receptor (TCR) on the CD8^+^ T-cells (Figure 1) for adaptive immune reactions. To evaluate the efficiency of HDEA@EVAT as a vaccination tool, we synthesized different types of nanoparticles (Table 1).

**Figure 2:**
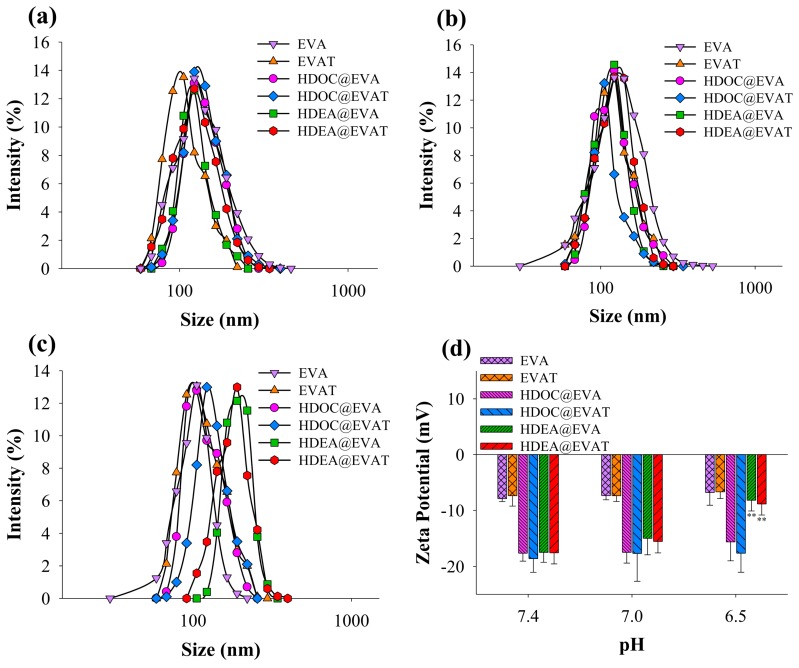
Particle size distributions of extracellular vesicles (EVs) at pH values of (**a**) 7.4; (**b**) 7.0; and (**c**) 6.5; (**d**) Zeta potential changes of EVs at pH values of 7.4, 7.0, or 6.5 (*n* = 3) (** *p* < 0.01 compared to pH of 7.4).

The loading contents of HDEA/HDOC, MPLA, and MUC1 were found to range from 15.9 to 17.7%, 3.3 to 3.7%, and 2.4 to 4.7%, respectively, when compared to the EV content. To study the pH-dependent physicochemical characteristics, the sizes and zeta potentials of EVs were measured at different pH values. The sizes of all particles were comparable at a pH of 7.4 (Figure 2a) or a pH of 7.0 (Figure 2b) and ranged between 100–140 nm. By contrast, the sizes of HDEA@EVAT and HDEA@EVA reached approximately 200 nm at a pH of 6.5 (Figure 2c) because they contain pH-sensitive HDEA conjugates which are protonated at the early endosomal pH of 6.5 [39,40], thus undermining the structure of EV particles and endosomes. As a result, the destabilized EVs were aggregated which led to size increases. However, since HDOC@EVA and HDOC@EVAT possess pH-irresponsive polysaccharide HDOC (Table 1), the mildly acidic pH of 6.5 did not affect their sizes, and thus led to solid structures. Similarly, while the surface charges of EVA, EVAT, HDOC@EVA, and HDOC@EVAT did not change at early endosomal pH values of 6.5 compared to those at pH values of 7.4 and 7.0, those of HDEA@EVA and HDEA@EVAT increased by approximately 9 mV at pH 6.5 compared to those at the neutral pHs (Figure 2d). These results suggest that the pH-responsive HDEA blocks in HDEA@EVA and HDEA@EVAT can physically change the formation of EVs in early endosomes that facilitates the endosomal escape.

### 3.2. MUC1 Release Analysis

**Figure 3:**
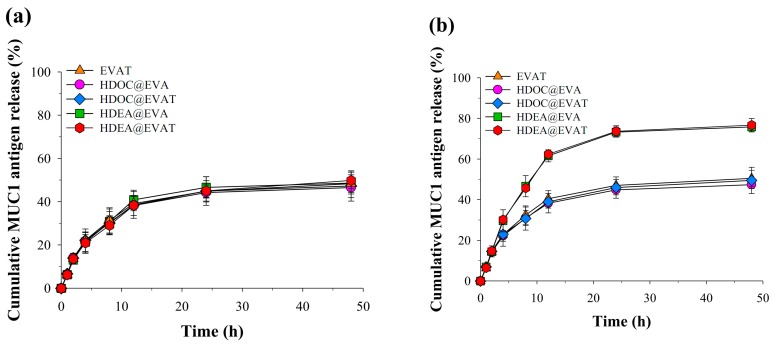
MUC1 antigen release kinetics from EVs at pH values of (**a**) 7.4 or (**b**) 6.5 for 48 h (*n* = 3).

MUC1 release from EVs was monitored at different pH values. They all reached a plateau (~51%) after 48 h at the physiological pH of 7.4 (Figure 3a). There was no difference among the EV groups regarding MUC1 secretions. To increase the MUC1 release, the EV should be destabilized. In agreement with the changes in size (Figure 2c), HDEA@EVA and HDEA@EVAT discharged MUC1 (~80%) more efficiently after 48 h at the endosomal pH of 6.5 (Figure 3b), thus suggesting that the MUC1 release from HDEA@EVA and HDEA@EVAT is involved in the endosomal pH. Furthermore, EVA yielded very similar results to the EVAT at both pH values (data not shown).

### 3.3. In Vitro Cellular Internalization Study

To validate biocompatibility, we used various concentrations of EVAT, HDOC@EVA, HDOC@EVAT, HDEA@EVA, or HDEA@EVAT for the treatments that used the human breast cancer cell line MCF–7 and RAW 264.7 cells (Figure S1, Supplementary Materials). The EVs were nontoxic in both cell lines. To selectively collect mature DCs, live DCs were sorted with the use of the cell viability marker acridine orange which stains nucleic acids [41]. Interestingly, differentiated DCs were larger than undifferentiated monocytes (Figure S2a, Supplementary Materials). To verify the maturation of DCs, we immunostained the DCs and immature monocytes with FITC–CD86 antibodies because the degree of DC maturation regulated the CD86 expression [12,13]. As expected, CD86 was upregulated on mature DCs (MFI is 120) compared to that on undifferentiated monocytes (MFI is 37.9) (Figure S2b–c). 

**Figure 4:**
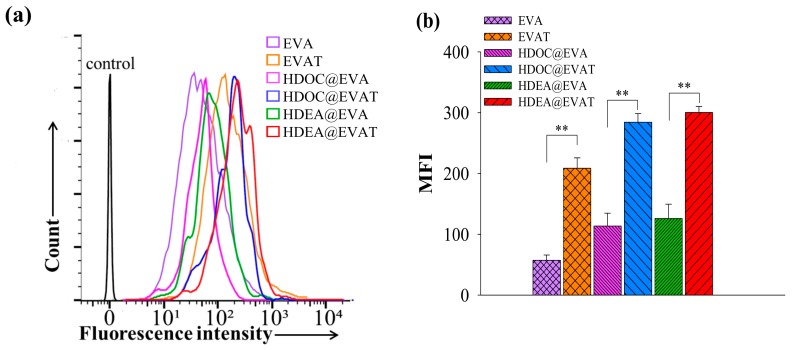
(**a**) Flow cytometry analyses or (**b**) mean fluorescence intensity (MFI) of DCs treated with fluorescent Ce6-labeled EVs after incubation for 4 h at 37 °C (*n* = 3).

**Figure 5:**
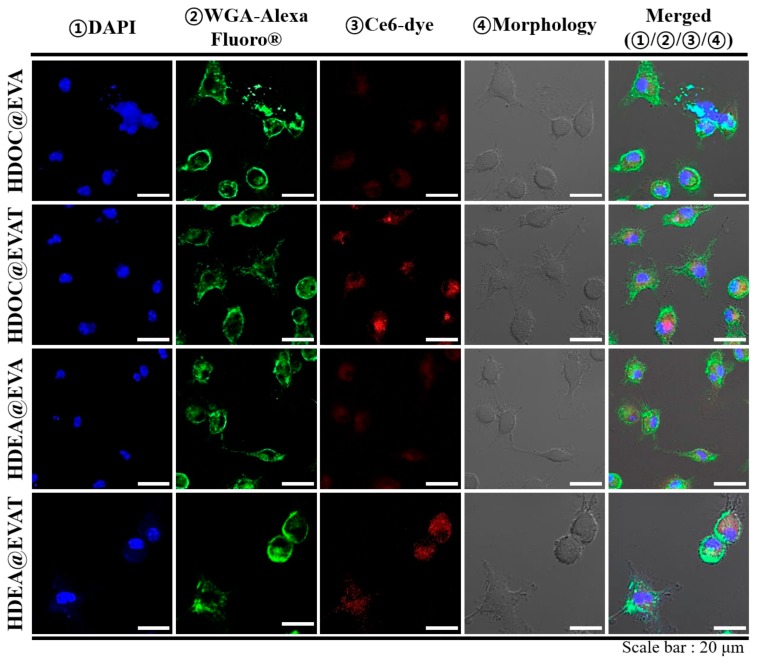
Confocal images of DCs treated with fluorescent Ce6-labeled EVs after incubation for 4 h at 37 °C.

Mature DCs express TLR4 after interactions with MPLA that contributes to LPS-involved immune responses [10,11,42]. To investigate how the MPLA conjugate on EVs affects the cellular uptake, fluorescent dye Ce6 was anchored to each EV particle, and it was administered into mature DCs. MPLA-loaded EVAT, HDOC@EVAT, and HDEA@EVAT (Table 1) respectively yielded intense fluorescence signals (Figure 4a) when the MFI values were equal to 208, 269, and 308 (Figure 4b). Conversely, EVA, HDOC@EVA, and HDEA@EVA, respectively yielded lower MFI values equal to 57, 92, and 149, owing to the deficiency of MPLA (Table 1). Notably, given that HDOC@EVAT and HDEA@EVAT contained the CD44 receptor-bound HA and CD44 receptors that are upregulated on DCs [33,34], they displayed better endocytosis rates than EVA. In a qualitative manner, confocal microscopy demonstrated that the intense red signals of Ce6 were detected from HDOC@EVAT- or HDEA@EVAT-treated DCs (Figure 5). These findings were consistent with the quantitative flow cytometry results (Figure 4). Taken together, EVs equipped with TLR4-interacted MPLA and CD44 receptor-recognized HA exhibited effective intracellular localization. In addition, HDOC@EVA and HDEA@EVA without MPLA led to low Ce6 red signals in the case of treated DCs. 

### 3.4. Extracellular Vesicle (EV)-Regulated DC Maturation and Antigen Presentation

**Figure 6:**
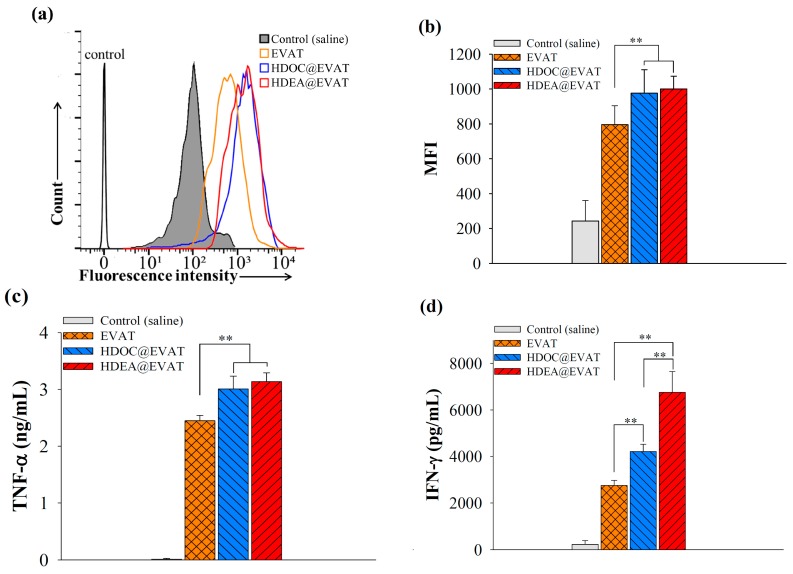
EVs induce DC maturation and CD8^+^ T-cell activation. (**a**) Flow cytometry and (**b**) MFI of DCs treated with control (saline) or EVs and with FITC–CD86 antibodies (*n* = 3); (**c**) TNF–α levels produced by DCs treated with control (saline), or EVs after incubation for 24 h at 37 °C (*n* = 3); (**d**) IFN–γ levels secreted by CD8^+^ T-cells interact with control (saline) or EV-treated mature DCs (*n* = 3).

To determine how different types of EVs regulate the maturation of DCs, we employed a mature DC-specific FITC–CD86 antibody [12,13]. The FITC–CD86 antibody-labeled EVs were incubated with DCs and flow cytometry revealed significantly higher uptake of EVAT, HDOC@EVAT, and HDEA@EVAT (Figure 6a). Quantitatively, MFI indicated that the EVAT, HDOC@EVAT, and HDEA@EVAT yielded approximately 4–5 times higher CD86 expressions than the controls (Figure 6b). This is because EVAT, HDOC@EVAT, and HDEA@EVAT contain TLR4-interacted MPLA that results in the DC maturation (Figure 1, upper enlarged circle). Markedly, in line with the Ce6-labeled EV data (Figure 4b), the HDOC@EVAT and HDEA@EVAT exhibited higher MFI than the EVAT, which is ascribed to the presence of HA, the ligand of the CD44 receptor on DCs. In addition, the ligand–receptor binding increased the efficiency of the TLR4 targeting by the MPLA. Given that EVA, HDOC@EVA, and HDEA@EVA lacked the MPLA, they did not stimulate DC maturation. This resulted in insignificant MFI values which were similar to the control (data not shown).

As a mature DC marker, the levels of TNF–α were identified. Whereas the saline control induced negligible TNF–α release, EVAT, HDOC@EVAT, and HDEA@EVAT, significantly promoted TNF–α secretion from DCs (Figure 6c). In addition, naïve EV or EVA did not stimulate TNF–α production from the DCs (data not shown). This suggests that the MPLA-associated EVs activated DC maturation. In particular, TNF–α production from the HDOC@EVAT or HDEA@EVAT application was higher than that from the EVAT, owing to the enhanced MPLA–TLR4 interaction that was assisted by the HA–CD44 receptor binding. 

To gain insights into the anticancer-involved immunological change attributed to the EV administration, IFN–γ levels from CD8^+^ T-cells were measured. MUC1 antigen in EVs was supposed to be delivered, released, and identified by TCR on the CD8^+^ T-cells to discharge the IFN–γ cytokine. In agreement with the TNF–α results (Figure 6c), the saline controls did not affect the IFN–γ secretion, but EVAT, HDOC@EVAT, and HDEA@EVAT significantly escalated the produced IFN–γ levels by CD8^+^ T-cells (Figure 6d). Among them, the HDEA@EVAT yielded the highest IFN–γ levels because it contained (a) MPLA used for TLR4-controlled DC maturation, (b) HA used for augmented MPLA–TLR4 binding, and (c) DEAP used for the pH-dependent endosomal escape of MUC1. This was followed by the effective antigen presentation of mature DCs to unprimed CD8^+^ T-cells. The antigen presentation led to the activation of the CD8^+^ T-cell that in turn led to the production of IFN–γ. Conversely, even though HDOC@EVAT and EVAT led to a remarkable IFN–γ secretion, the deficiency of pH-responsive DEAP in HDOC@EVAT, and the lack of pH-sensitivity and CD44 ligands in EVAT activated CD8^+^ T-cells to a lesser extent. Taking this into account, HDEA@EVAT is considered to be a potent DC-targeted anticancer vaccination agent. 

## 4. Conclusions

The EV-based anticancer vaccine nanoparticle HDEA@EVAT was designed and developed by conjugating pH-sensitive DEAP, CD44 receptor-bound polysaccharide HA, TLR4-associated MPLA, and cancer-involved antigen MUC1. This nanoparticle showed pH-dependent physicochemical characteristics and MUC1 release responses, and interacted with DC for effective cellular internalization. Regarding its intracellular behavior, it effectively escaped from endosomes and carried MUC1 into the DC cytosol. MUC1 was processed and presented to CD8^+^ T-cells, and primed them for advanced cancer immunity. Collectively, this approach provided a promising vaccine delivery system that was used to trigger strong T-cell responses against cancers. 

## Figures and Tables

**Table 1:** Components of used particles. Y indicates the presence and N indicates the absence of these particles.

Name	EV	MUC1 (Antigen)	MPLA (TLR4 Ligand)	HDOC	HDEA
EVA	Y	Y	N	N	N
EVAT	Y	Y	Y	N	N
HDOC@EVA	Y	Y	N	Y	N
HDOC@EVAT	Y	Y	Y	Y	N
HDEA@EVA	Y	Y	N	N	Y
HDEA@EVAT	Y	Y	Y	N	Y

## Supplementary Materials

The following are available online at http://www.mdpi.com/1999-4923/11/2/54/s1, Figure S1: Cell viability test of (**a**) MCF-7 cells or (**b**) RAW 264.7 cells after 24 h treatment with EVs at 37 °C (*n* = 7). Figure S2: (**a**) Confocal images of monocytes and DCs stained with the acridine orange dye. Monocytes differentiated into DCs by incubating them with GM–CSF and IL–4 for 7 d. Flow cytometry analyses and dot plots of (**b**) monocytes or (**c**) DCs treated with FITC–CD86 antibodies. Figure S3: Transmission electron microscope (TEM) image of HDEA@EVAT at pH 7.4.
