# Supplementary material for: Dendritic Cell-Targeted pH-Responsive Extracellular Vesicles for Anticancer Vaccination
Source: Pharmaceutics. 2019 Jan 27;11(2):54. doi: 10.3390/pharmaceutics11020054 (PMC6410067; doi:10.3390/pharmaceutics11020054)
Supplement: Supplementary file 1 [file pharmaceutics-11-00054-s001.pdf]

pharmaceutics

MDPI

Supplementary Materials: Dendritic Cell-targeted pH-responsive Extracellular Vesicles for Anticancer Vaccination

Hyuk Lee, Hongsuk Park, Hyeong Sup Yu, Kun Na, Kyung Taek Oh and Eun Seong Lee

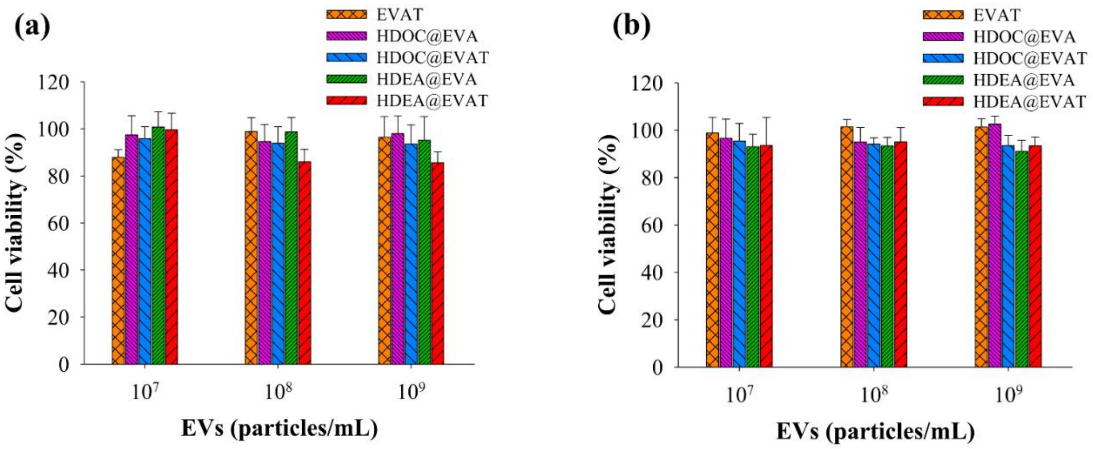

Figure S1. Cell viability test of (a) MCF-7 cells or (b) RAW 264.7 cells after 24 h treatment with EVs at 37 °C (n=7).

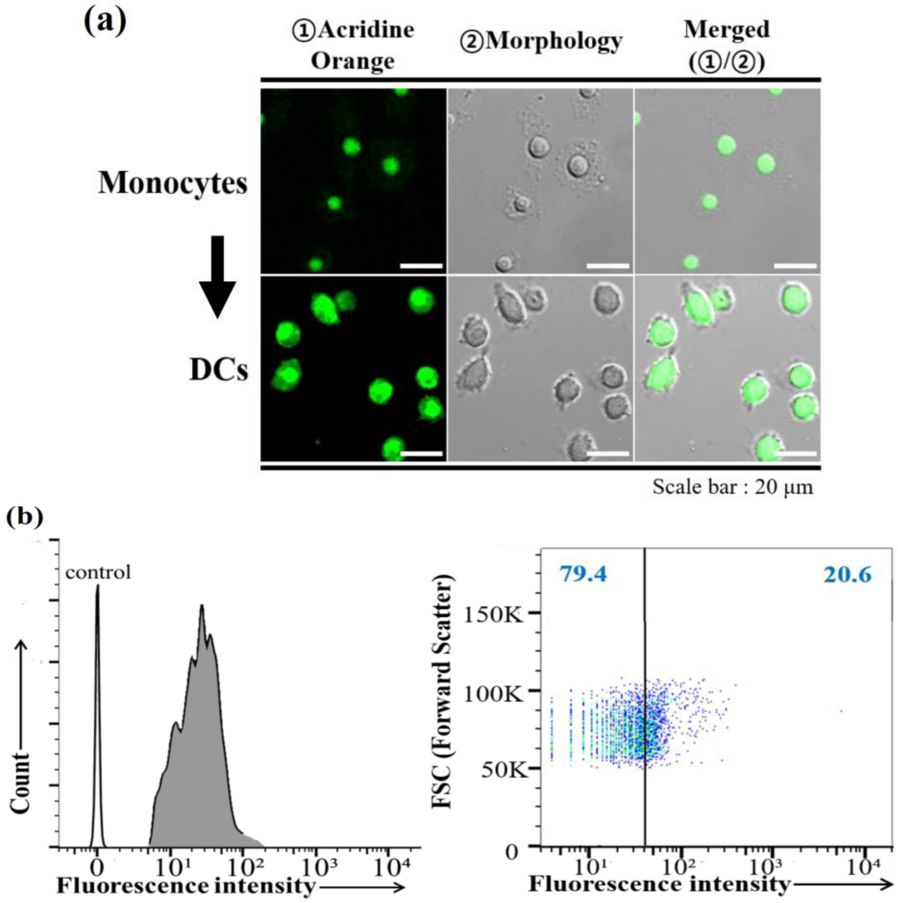

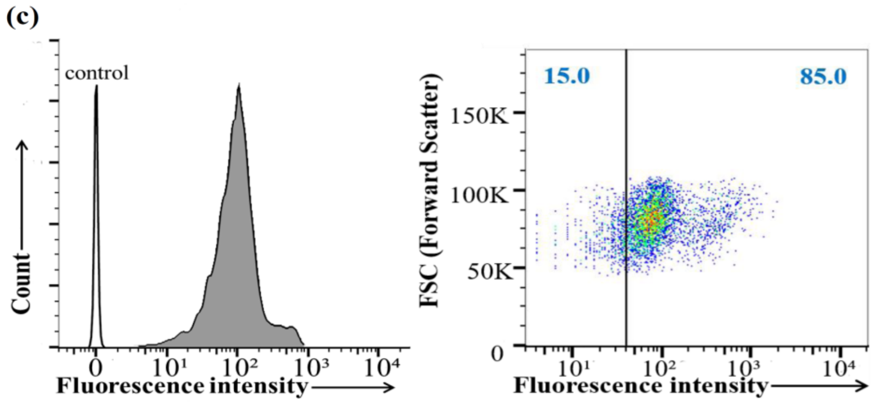

Figure S2. (a) Confocal images of monocytes and DCs stained with the acridine orange dye. Monocytes differentiated into DCs by incubating them with GM–CSF and IL–4 for 7 d. Flow cytometry analyses and dot plots of (b) monocytes or (c) DCs treated with FITC–CD86 antibodies.

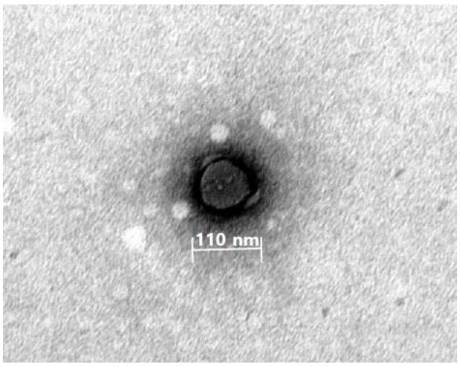

Figure S3. Transmission electron microscope (TEM) image of HDEA@EVAT at pH 7.4.
